# Functional Outcome and Donor Site Morbidity Following Posterior Cruciate Ligament (PCL) Reconstruction Using Peroneus Longus Tendon Autograft: A Prospective Cohort Study

**DOI:** 10.7759/cureus.108022

**Published:** 2026-04-30

**Authors:** Hiranya Kumar, Shuchindra R Chowdhary, B. A Pradeep, Nishanth J Reddy, Shivaraj Nadagouda, Srinivasa Somasundara

**Affiliations:** 1 Orthopaedics and Trauma, Vydehi Institute of Medical Sciences and Research Centre, Bengaluru, IND; 2 Orthopaedics, Vydehi Institute of Medical Sciences and Research Centre, Bangalore, IND

**Keywords:** a prospective cohort study, ligamentous knee injury, pcl reconstruction, posterior cruciate ligament (pcl) injuries, sports injury surgery

## Abstract

Background

Posterior cruciate ligament (PCL) injuries present significant challenges in orthopaedic management. The peroneus longus tendon (PLT) has emerged as a potential autograft source; however, its role in PCL reconstruction remains underexplored.

Methods

A prospective observational study was conducted on 25 patients aged 18-50 years with isolated PCL injuries who underwent arthroscopic single-bundle reconstruction using ipsilateral PLT autograft. Functional outcomes were assessed using the Tegner Lysholm Knee Score, while donor site morbidity was evaluated using the Foot and Ankle Disability Index (FADI) and the American Orthopedic Foot and Ankle Society (AOFAS) score at one, three, and six months postoperatively.

Results

The mean Lysholm score improved significantly from 48.60 ± 7.77 preoperatively to 83.84 ± 8.13 at six months (p < 0.001). FADI and AOFAS scores showed an initial postoperative decline followed by near-complete recovery at six months (p < 0.001). Complications occurred in 16% of patients.

Conclusion

PLT autograft provides significant functional improvement with minimal donor site morbidity, supporting its use as a viable alternative graft option in PCL reconstruction.

## Introduction

The posterior cruciate ligament (PCL) is the largest and strongest intra-articular ligament of the knee and serves as the primary restraint to posterior tibial translation [1]. It contributes approximately 95% of resistance to posterior tibial displacement at 30-90° of knee flexion [2]. The incidence of PCL injuries ranges from 1% to 44% of all acute knee injuries, most commonly resulting from road traffic accidents and sports-related trauma [3,4].

Anatomically, the PCL originates from the lateral surface of the medial femoral condyle and inserts into the posterior tibial plateau. It consists of two functionally interdependent bundles-the anterolateral and posteromedial bundles-that act synergistically throughout knee motion [2,5,6]. Clinically, the posterior drawer test remains the most reliable diagnostic tool, with high sensitivity and specificity reported in the literature. MRI serves as a confirmatory imaging modality with excellent diagnostic accuracy, while stress radiography may be used to quantify posterior tibial translation [7,8].

Management depends on injury severity. Non-operative treatment is recommended for grade I and grade II injuries, whereas surgical reconstruction is indicated in symptomatic grade III injuries [5,6]. Graft selection plays a critical role in surgical outcomes. Bone-patellar tendon-bone (BPTB) autografts are associated with anterior knee pain, while hamstring tendon grafts may result in reduced strength and unpredictable graft size [9,10].

The peroneus longus tendon (PLT) has recently gained attention as an alternative graft. Biomechanical studies demonstrate tensile strength comparable to that of hamstring tendons [11]. Meta-analyses of ACL reconstruction have shown similar functional outcomes with reduced donor site morbidity [12,13]. However, literature regarding PLT use in PCL reconstruction remains limited. Setyawan et al. demonstrated promising outcomes in a small cohort [14].

## Materials and methods

Study design and settings

This was a prospective observational study conducted in the Department of Orthopaedics at Vydehi Institute of Medical Sciences and Research Centre, Bengaluru, India, over a period of two years. This study was conducted to evaluate functional outcomes and donor site morbidity following arthroscopic PCL reconstruction using PLT autograft.

Sample size calculation

The sample size was calculated with alpha (α) = 0.05, beta (β) = 0.2 (power = 80%), mean of difference (δ) = 30.99, and standard deviation of difference (σ) = 14.0. The minimum required sample size was determined to be 25 patients.

Study population

A total of 25 patients who presented with isolated PCL injuries and met the eligibility criteria were enrolled consecutively. Patients were selected based on predefined inclusion and exclusion criteria, including isolated PCL injuries and the absence of multi-ligament involvement. Eligible patients included adults aged 18-50 years with isolated PCL injuries confirmed clinically and radiologically. The inclusion and exclusion criteria used for patient selection are summarized in Table 1.

**Table 1:** Inclusion and exclusion criteria

Inclusion Criteria	Exclusion Criteria
Age between 18 and 50 years	Inflammatory arthropathy of the knee joint
High-risk knee injuries including sports injuries	Active infective conditions of the knee
Isolated PCL injuries confirmed by clinical examination and MRI	Inflammatory conditions of the ankle joint
Willingness to provide written informed consent	Multi-ligament injury of the knee
	Refusal to consent

Pre-operative evaluation

All enrolled patients underwent a standardised pre-operative assessment including detailed history, clinical examination, and routine investigations such as complete blood count, blood grouping, random blood sugar, renal function tests, serum electrolytes, erythrocyte sedimentation rate (ESR), C-reactive protein (CRP), electrocardiograph (ECG), chest radiograph, knee radiographs, MRI, and urine analysis. Written informed consent was obtained from all participants.

Outcome measures

Functional outcomes were assessed using the Tegner Lysholm Knee Score [15]. Donor site morbidity was evaluated using the Foot and Ankle Disability Index (FADI) [16] and the American Orthopaedic Foot and Ankle Society (AOFAS) ankle-hindfoot score [17].

Surgical technique

All procedures were performed under appropriate anaesthesia by a single experienced surgical team. The patient was positioned supine with a tourniquet applied. Graft harvesting: The peroneus longus tendon was harvested via a 3 cm incision proximal to the lateral malleolus. The distal stump was sutured to the peroneus brevis tendon, and the graft was stripped proximally. Arthroscopic PCL reconstruction: Standard arthroscopic portals were established. Femoral and tibial tunnels were created, and the graft was fixed using a cortical button and an interference screw with the knee at 90° flexion.

Post-operative rehabilitation

A standard rehabilitation protocol was followed, including early ankle mobilisation and gradual progression to weight-bearing and range of motion exercises. Patients were evaluated at one, three, and six months using Lysholm, FADI, and AOFAS scores. Complications were recorded at each follow-up.

Statistical analysis

Data were analysed using SPSS software, version 25 (IBM Corp., Armonk, NY). Quantitative variables were expressed as mean ± standard deviation, and qualitative variables as frequencies and percentages. Repeated measures analysis was used to assess changes over time. A p-value < 0.05 was considered statistically significant.

## Results

Demographic characteristics

A total of 25 patients who underwent arthroscopic PCL reconstruction with PLT autograft were included. The distribution of age groups is presented in Table 2. The mean age of the study population was 33.04 ± 6.85 years. The age groups 21-30 years and 31-40 years were equally represented, each comprising 11 (44%) patients, while only three (12%) patients belonged to the 41-50 years age group.

**Table 2:** Distribution of age groups

Age Group (years)	Frequency, n (%)
21–30	11 (44.00)
31–40	11 (44.00)
41–50	3 (12.00)
Total	25 (100.00)
Mean ± SD	33.04 ± 6.85 years

The sex distribution is shown in Table 3. A marked male predominance was observed, with 21 (84%) males and four (16%) females.

**Table 3:** Distribution by sex

Sex	Frequency, n (%)
Female	4 (16.00)
Male	21 (84.00)
Total	25 (100.00)

The mechanism of injury is presented in Table 4. Sports injuries were the most common mechanism (56%), which included both contact and non-contact injuries such as pivoting, sudden deceleration, and directional changes.

**Table 4:** Mechanism of injury

Mechanism of Injury	Frequency, n (%)
Sports Injury	14 (56.00)
Twisting injuries & slip and fall	7 (28.00)
Road Traffic Accident	4 (16.00)
Total	25 (100.00)

The distribution of injury by side is presented in Table 5. Right-sided injuries were slightly more prevalent, affecting 14 (56%) patients, compared to left-sided injuries in 11 (44%) patients.

**Table 5:** Site of injury

Site Injured	Frequency, n (%)
Right (Dextra)	14 (56.00)
Left (Sinistra)	11 (44.00)
Total	25 (100.00)

Functional outcome assessment

The Tegner Lysholm Knee Score demonstrated progressive and statistically significant improvement from the pre-operative period through all post-operative follow-up time points, as shown in Table 6. The mean score improved from 48.60 ± 7.77 pre-operatively to 63.84 ± 8.13 at one month, 73.84 ± 8.13 at three months, and 83.84 ± 8.13 at six months post-operatively, representing a mean overall improvement of 35.24 points (p < 0.001).

**Table 6:** Tegner Lysholm Knee Scores at pre-operative and post-operative time points

Time Point	Mean	Std. Deviation	p-value
Pre-operative	48.60	7.77	
Post-op (1 month)	63.84	8.13	
Post-op (3 months)	73.84	8.13	
Post-op (6 months)	83.84	8.13	< 0.001

The categorical classification of the Tegner Lysholm scores further demonstrated progressive recovery, as shown in Table 7. Pre-operatively, all 25 (100%) patients were classified as “Poor.” At one month, 12 (48%) remained “Poor” while 13 (52%) transitioned to “Fair.” By three months, four (16%) remained “Poor,” 19 (76%) were classified as “Fair,” and two (8%) achieved “Good.” At six months, no patients remained “Poor”; 12 (48%) were “Fair,” eight (32%) were “Good,” and five (20%) were “Excellent” (p < 0.001).

**Table 7:** Tegner Lysholm Knee Score classification at various time points, n (%)

Time Point	Poor	Fair	Good	Excellent
Pre-operative	25 (100)	0 (0)	0 (0)	0 (0)
1 Month Post-op	12 (48)	13 (52)	0 (0)	0 (0)
3 Months Post-op	4 (16)	19 (76)	2 (8)	0 (0)
6 Months Post-op	0 (0)	12 (48)	8 (32)	5 (20)

Donor site morbidity assessment

The FADI scores revealed transient donor site morbidity followed by near-complete recovery, as shown in Table 8. The mean pre-operative FADI score was 93.20 ± 7.79, which declined to 83.40 ± 7.79 at one month post-operatively. Scores improved to 88.40 ± 7.79 at three months and 92.60 ± 7.00 at six months, nearly returning to baseline (p < 0.001).

**Table 8:** FADI scores at pre-operative and post-operative time points FADI: Foot and Ankle Disability Index.

Time Point	Mean	Std. Deviation	p-value
Pre-operative	93.20	7.79	
Post-op (1 month)	83.40	7.79	
Post-op (3 months)	88.40	7.79	
Post-op (6 months)	92.60	7.00	< 0.001

The AOFAS scores followed a similar trajectory, as shown in Table 9. The pre-operative mean of 96.40 ± 7.79 decreased to 88.40 ± 7.79 at one month and improved to 95.60 ± 5.55 at six months, indicating near-complete recovery of ankle function (p < 0.001).

**Table 9:** AOFAS scores at pre-operative and post-operative time points AOFAS: American Orthopedic Foot and Ankle Society.

Time Point	Mean	Std. Deviation	p-value
Pre-operative	96.40	7.79	
Post-op (1 month)	88.40	7.79	
Post-op (3 months)	92.60	7.00	
Post-op (6 months)	95.60	5.55	< 0.001

The categorical AOFAS classification at each time point is shown in Table 10. At six months, 21 (84%) patients were classified as “Excellent,” while four (16%) were “Good,” with no patients in the “Fair” category (p < 0.001).

**Table 10:** AOFAS score classification at various time points, n (%)

Time Point	Fair	Good	Excellent
Pre-operative	0 (0)	17 (68)	8 (32)
1 Month Post-op	1 (4)	11 (44)	13 (52)
3 Months Post-op	0 (0)	6 (24)	19 (76)
6 Months Post-op	0 (0)	4 (16)	21 (84)

The distribution of post-operative complications is presented in Table 11. The overall complication rate was four out of 25 patients (16%). Two (8%) patients developed infections managed with antibiotics, and two (8%) reported persistent ankle pain. The remaining 21 (84%) patients had no complications.

**Table 11:** Distribution of complications

Complication	Frequency, n (%)
Infection	2 (8.00)
Persistent ankle and foot pain	2 (8.00)
None	21 (84.00)
Total	25 (100.00)

## Discussion

The present study demonstrates a well-structured longitudinal assessment of outcomes, allowing evaluation of both early and progressive functional recovery following PCL reconstruction. The predominance of sports-related injuries, particularly involving pivoting and sudden directional changes, highlights the role of rotational stress in PCL injury mechanisms. Common mechanisms include dashboard injuries, hyperflexion trauma, and sports-related pivoting injuries involving a planted foot with sudden rotational forces.

This prospective study demonstrates that arthroscopic PCL reconstruction using PLT autograft results in significant and progressive improvement in knee function over six months, accompanied by minimal, largely transient donor site morbidity. These findings are consistent with recent literature evaluating peroneus longus tendon autografts. Gök et al. reported comparable functional outcomes and minimal donor site morbidity in ACL reconstruction using PLT autografts [18]. Similarly, Punnoose et al. demonstrated superior graft diameter and reduced morbidity with PLT compared to hamstring tendon autografts [19]. Goyal et al. also reported favourable outcomes with full-thickness PLT autografts in complex and revision ligament reconstruction cases, highlighting its versatility [20]. Recent studies have also demonstrated improved functional outcomes and preserved muscle function in PLT-based PCL reconstruction. 

The observed Lysholm improvement (48.60 to 83.84) is consistent with previously reported PLT-based reconstructions and comparable to outcomes with traditional grafts. The progressive shift from predominantly “Poor” pre-operative scores to “Fair-Excellent” categories at six months underscores meaningful clinical recovery. Donor site morbidity, as assessed by FADI and AOFAS, followed an expected early post-harvest decline with near-complete recovery by six months. The minimal difference between baseline and final AOFAS scores suggests preserved ankle function, likely attributable to distal tenodesis of the PLT to the peroneus brevis. The preservation of ankle function observed in this study may be attributed to distal tenodesis of the peroneus longus tendon to the peroneus brevis. This technique helps maintain eversion strength and ankle stability, thereby minimizing donor site morbidity. Previous biomechanical and clinical studies have also demonstrated that distal tenodesis compensates effectively for the harvested tendon, allowing near-normal functional recovery.

The overall complication rate (16%) was acceptable, with infections managed conservatively and no neurovascular injuries or ankle instability observed. These findings support the safety profile of PLT harvesting. Clinical implications: PLT offers (i) adequate and consistent graft diameter, (ii) avoidance of knee donor site morbidity, and (iii) preservation of ankle function, advantages particularly relevant in populations requiring frequent kneeling or squatting.

Strengths

This study has several strengths, including its prospective design, standardized surgical technique performed by a single experienced team, and the use of validated outcome measures (Tegner Lysholm, FADI, and AOFAS scores). Additionally, the simultaneous evaluation of both knee function and donor site morbidity provides a comprehensive clinical assessment.

Limitations and future directions

However, the study has limitations, including a small sample size (n = 25), the absence of a control or comparison group, and a short follow-up duration of six months. Furthermore, objective measures of knee stability, such as stress radiography, were not included, and sport-specific functional outcomes were not extensively analyzed. Future directions: randomized comparative trials versus hamstring/BPTB grafts, longer follow-up, and incorporation of objective stability measures are needed to establish definitive recommendations.

## Conclusions

This prospective study demonstrates that arthroscopic posterior cruciate ligament reconstruction using the peroneus longus tendon autograft produces significant and progressive functional improvement in knee function over a six-month follow-up period. Donor site morbidity was minimal and largely transient, with ankle function scores returning to near pre-operative baseline levels by the final follow-up, and the majority of patients experiencing no post-operative complications. These findings are consistent with previously published studies, and while the findings support the peroneus longus tendon as a viable graft option with favorable functional outcomes and minimal donor site morbidity, the results should be interpreted in light of the study’s limitations, including small sample size and short follow-up duration. Larger, randomized studies with longer follow-up are required to validate these findings. Further research in the form of larger, multi-centre, randomised controlled trials with longer follow-up periods is warranted to validate these findings, compare PLT outcomes with conventional graft sources, and establish standardised techniques for PLT harvesting and application in PCL reconstruction.
